# Factors Associated With Multivessel Coronary Artery Involvement in Patients With Coronary Artery Disease: A Retrospective Observational Study

**DOI:** 10.7759/cureus.113064

**Published:** 2026-07-21

**Authors:** Naeem A Alshoaibi, Taha K Tasji, Sultan Gharib, Abrahem M Alsherbini, Arwa F Lardhi

**Affiliations:** 1 Cardiology, King Abdulaziz University Hospital, Jeddah, SAU; 2 Internal Medicine, King Fahad Armed Forces Hospital, Ministry of Defense Health Services, Jeddah, SAU; 3 Cardiology, King's College Hospital London, Jeddah, SAU; 4 Cardiology, Doctor Soliman Fakeeh Hospital, Fakeeh College, Jeddah, SAU

**Keywords:** advanced age, coronary artery disease, multivessel coronary artery involvement, obesity, pci

## Abstract

Objectives: Coronary artery disease can affect a single vessel, two vessels, or multiple vessels. Percutaneous coronary intervention (PCI) has shown significant results in improving myocardial perfusion and reducing reinfarction; however, a large proportion of PCI patients are at risk of increased complications due to multivessel coronary artery involvement and other clinical characteristics, such as obesity and age. This study aimed to identify demographic and clinical factors associated with multivessel coronary artery disease, double- or triple-vessel involvement, among patients undergoing PCI, and to compare these characteristics between patients undergoing PCI for double-vessel and triple-vessel disease. Materials and methods: This retrospective observational study was conducted at Doctor Soliman Fakeeh Hospital (DSFH), Jeddah, Saudi Arabia, between January 2019 and December 2024. This study included adult patients diagnosed with coronary artery disease who underwent PCI at DSFH. Patients were categorized into single-vessel PCI, double-vessel PCI, or triple-vessel PCI groups. Results: A total of 1,329 patients were included in the study, with 978 undergoing single-vessel PCI, 324 undergoing double-vessel PCI, and 27 undergoing triple-vessel PCI. Patients aged ≥65 years had nearly twice the odds of undergoing multivessel PCI compared with those <45 years (adjusted odds ratio (aOR) 1.90, 95% CI 1.17-3.09, p = 0.009). The distribution of body mass index categories differed significantly (p < 0.001). Obese patients were more than two-and-a-half times more susceptible to having multivessel involvement compared to patients with normal weight (aOR 2.62, 95% CI 1.77-3.88, p < 0.001). Advanced age and obesity were significant factors associated with PCI after limited adjustment for multivessel coronary artery disease, whereas the overweight status was not associated with increased odds. Conclusions: Although several factors can cause coronary artery disease, obesity and advanced age were found to be significant factors associated with multivessel coronary artery disease requiring PCI. This study provides additional evidence supporting the association between advanced age, obesity, and multivessel coronary artery disease among patients undergoing PCI.

## Introduction

Cardiovascular diseases are a broad spectrum of conditions, including hypertension, stroke, coronary artery disease, and congenital heart disease [1]. Coronary artery disease is considered a leading cause of death globally. For instance, the prevalence of cardiovascular diseases has doubled from 271 million in 1990 to 523 million in 2019, with deaths reaching 19.7 million that year. Moreover, disability-adjusted life years and the number of years of life lost also doubled from 17.7 million in 1990 to 34.4 million in 2019 [2]. At the same time, the global burden of ischemic heart disease and coronary artery disease reached 197 million in 2019, with deaths reaching 9.14 million [2]. Multiple risk factors can cause coronary artery disease, such as hypertension, diabetes, hypercholesterolemia, smoking, and genetics [1].

Coronary artery disease can affect a single vessel, two vessels, or multiple vessels. Additionally, non-obstructive coronary artery disease is often associated with long-term complications, especially when three vessels are affected. When the number of affected vessels increases, the frequency of five-year major adverse cardiac and cerebrovascular events (MACCE) increases [3]. For instance, patients with three diseased vessels are twice as likely to experience MACCE and stroke, in addition to being 15 times more prone to myocardial infarction compared to patients without non-obstructive diseased vessels [3].

Percutaneous coronary intervention (PCI) refers to a range of procedures that include reopening an obstructed coronary artery, aiming to improve myocardial perfusion without the need to perform coronary artery bypass surgery [4]. Additionally, it significantly improves the symptoms of stable angina and the prognosis of acute coronary syndromes [4]. Although PCI has shown significant results in improving myocardial perfusion, reducing reinfarction and stroke, and lowering death rates, a large proportion of PCI patients are at risk of increased complications due to the presence of multivessel coronary artery involvement [5]. Multivessel coronary artery involvement is present in about 40% to 50% of patients with acute myocardial infarction; therefore, determining the extent of coronary artery disease involvement is beneficial in patients undergoing PCI to predict the procedural outcome and mitigate the possible complications [6,7]. Moreover, other characteristics, such as obesity, hypertension, age, and diabetes, can affect the success of PCI [8]. High blood pressure, high fasting plasma glucose levels, obesity, high serum triglycerides, and reduced levels of high-density lipoprotein cholesterol (HDL-C) are prevalent in about 39.2% of patients with coronary artery disease undergoing PCI [9]. For instance, higher mean years of age, diabetes, and hypertension were found to be significantly associated with poorer outcomes and increased mortality in patients with multivessel disease receiving PCI [10]. Additionally, obesity substantially contributes to the development of type 2 diabetes due to multiorgan insulin resistance and the decline in the insulin secretory function of β-cells, in addition to accelerating the atherosclerotic changes, hence contributing to the development of cardiovascular disease [11].

This retrospective observational study aims to identify demographic and clinical factors associated with multivessel coronary artery disease and double- or triple-vessel involvement among patients undergoing PCI and to compare demographic and clinical characteristics between patients undergoing double-vessel and triple-vessel PCI. Additionally, it aims to fill the gap in the existing literature that discusses the demographic and clinical factors between patients undergoing double-vessel and triple-vessel PCI.

## Materials and methods

Study design and settings

This is a retrospective observational study conducted at Doctor Soliman Fakeeh Hospital (DSFH), Jeddah, Saudi Arabia, between January 2019 and December 2024. The study was conducted on patients diagnosed with coronary artery disease who underwent PCI.

Study population

This study included 1,329 adult patients (aged ≥18 years) diagnosed with coronary artery disease who underwent PCI at DSFH between January 2019 and December 2024. All adult patients (≥18 years) who underwent left cardiac catheterization followed by PCI during the study period were eligible for inclusion. Patients were categorized according to angiographic findings and the number of coronary vessels treated during PCI into single-vessel PCI, double-vessel PCI, or triple-vessel PCI groups.

Cases with incomplete procedural data or missing information on vessel involvement were excluded because these variables were essential for defining the primary study outcome. A complete-case analysis approach was adopted because the missing information could not be reliably recovered or imputed from the retrospective electronic medical records.

Definition of study outcome

The primary study outcome was angiographically confirmed multivessel coronary artery disease. Multivessel CAD was defined as the involvement of two or more major epicardial coronary arteries on coronary angiography. As all patients in this study underwent PCI, they were categorized according to the number of treated vessels into single-vessel PCI, double-vessel PCI, or triple-vessel PCI.

Data collection

Demographic, clinical, laboratory, and procedural data were extracted retrospectively from electronic medical records using a standardized data abstraction form. All data were anonymous before the analysis.

Statistical analysis

All statistical analyses were performed using SPSS Statistics version 26 (IBM Corp. Released 2019. IBM SPSS Statistics for Windows, Version 26.0. Armonk, NY: IBM Corp.). Continuous variables were summarized using means and standard deviations for normally distributed data and medians with interquartile ranges for non-normally distributed data. Categorical variables were presented as frequencies and percentages. Comparisons between groups were conducted using the chi-square test or Fisher’s exact test for categorical variables and the independent samples t-test or Mann-Whitney U test for continuous variables, depending on data distribution. Differences in baseline characteristics among patients undergoing single-vessel, double-vessel, and triple-vessel PCI were assessed using the chi-square test or Fisher's exact test, as appropriate. To identify factors associated with angiographically confirmed multivessel coronary artery disease, multivariable logistic regression analysis was performed. Patients with double- or triple-vessel PCI (representing multivessel CAD) were compared with those undergoing single-vessel PCI.

Variable selection for the multivariable model was performed a priori based on the primary study objective and the availability of complete data. Age, sex, and BMI were selected because they were considered clinically relevant baseline demographic variables and were consistently available for all patients. Other established predictors of coronary artery disease severity, including smoking status, medication use, renal function, acute coronary syndrome presentation, and some clinical variables, were incompletely documented in the retrospective database and therefore could not be reliably incorporated into the multivariable model.

Adjusted odds ratios (aORs) with 95% confidence intervals (CIs) were reported. All statistical tests were two-tailed, and a p-value <0.05 was considered statistically significant.

Ethical consideration

The Institutional Review Board (IRB) of DSFH approved this study on 15 November 2023 (approval number: 518/IRB/2023). All procedures conducted in this study were in accordance with the ethical standards of the IRB. As this was a retrospective study using anonymized medical records, no direct patient contact occurred, and individual consent was waived in compliance with IRB guidelines. Both the confidentiality and privacy of the collected data were strictly preserved throughout the study.

## Results

During the study period, 1,378 patients were screened for eligibility. Of these, 49 patients were excluded because of incomplete procedural records or missing information on coronary vessel involvement, leaving 1,329 patients for the final analysis.

The baseline characteristics across the single-, double-, and triple-vessel PCI groups are shown in Table 1. The mean age increased modestly from 59.11 ± 11.88 years in the single-vessel group to 61.18 ± 10.83 years in the double-vessel group and 62.33 ± 9.09 years in the triple-vessel group. The age groups of 55-64 and 65-74 had the highest rates of all types of PCI. Males represented 81.9% of the single-vessel, 81.5% of the double-vessel, and 85.2% of the triple-vessel PCI groups. Lower BMI categories (normal and overweight) were predominantly observed in the single-vessel group. The distribution of procedures across years (2019-2024) was similar among the groups.

**Table 1:** Baseline characteristics of all patients by PCI type BMI category definitions: normal weight: 18.5-24.9 kg/m², overweight: 25.0-29.9 kg/m², obese: ≥30.0 kg/m² BMI: body mass index, PCI: percutaneous coronary intervention, SD: standard deviation

Variable	Single vessel PCI (n = 978)	Double vessel PCI (n = 324)	Triple vessel PCI (n = 27)
Age, mean ± SD	59.11 ± 11.875	61.18 ± 10.834	62.33 ± 9.094
Age group, n (%)			
<45	110 (11.2)	25 (7.7)	1 (3.7)
45-54	195 (19.9)	61 (18.8)	4 (14.8)
55-64	331 (33.8)	106 (32.7)	10 (37)
65-74	230 (23.5)	101 (31.2)	10 (37)
>75	112 (11.5)	31 (9.6)	2 (10.9)
Gender			
Male	801 (81.9)	264 (81.5)	23 (85.2)
Female	177 (18.1)	60 (18.5)	4 (14.8)
BMI, mean ± SD (kg/m²)	30.4408 ± 6.22	30.2765 ± 5.54	31.4408 ± 6.64
BMI group, n (%)			
Normal weight	175 (17.9)	36 (11.1)	0 (0.0)
Overweight	342 (35)	74 (22.8)	0 (0.0)
Obese	461 (47.1)	214 (66.0)	27 (100)
Year of procedure, n (%)			
2019	154 (15.7)	47 (14.5)	6 (22.2)
2020	224 (22.9)	83 (25.6)	5 (18.5)
2021	178 (18.2)	49 (15.1)	5 (18.5)
2022	169 (17.3)	48 (14.8)	5 (18.5)
2023	175 (17.9)	74 (22.8)	4 (14.8)
2024	78 (8)	23 (7.1)	2 (7.4)

Among patients with multivessel disease, the baseline characteristics of those undergoing double-vessel PCI and triple-vessel PCI were largely similar across most variables. The mean age did not differ significantly between groups (61.23 ± 10.86 vs. 62.33 ± 9.09 years, p = 0.597), and the distribution across age categories was comparable (p = 0.862), with the majority of patients in both groups falling within the 55-74 year range. Gender distribution was also nearly identical (male: 81.5% vs. 81.8%; p = 0.632). BMI categories showed a significant difference (p < 0.001): all triple-vessel PCI patients were classified as obese (100%), whereas the double-vessel group included a broader range of BMI categories, including normal weight (11.1%), overweight (22.8%), and obese (66%).

The distribution of procedures by year did not differ meaningfully between groups (p = 0.758), suggesting no temporal trend influencing PCI severity within the multivessel cohort. Lipid parameters, including total cholesterol, triglycerides, HDL-C, and LDL-C, were all comparable between groups, with no statistically significant differences. Similarly, diabetes-related indicators, including diabetes prevalence (58% vs. 63%, p = 0.617); HbA1c levels (6.97 vs. 7.43, p = 0.491); prediabetes status (p = 0.884); and controlled vs. uncontrolled diabetes (p = 0.648 and p = 0.288, respectively), did not differ significantly between groups. Hypertension was slightly more common in triple-vessel PCI patients (81.5% vs. 67.3%), but this difference did not reach statistical significance (p = 0.127) (Table 2).

**Table 2:** Baseline characteristics of multivessel PCI Statistical tests used: chi-square test, Fisher’s exact test, independent samples t-test, and Mann–Whitney U test. * Significant p-value <0.05. PCI: percutaneous coronary intervention, BMI: body mass index, HDL-C: high-density lipoprotein cholesterol, LDL-C: low-density lipoprotein cholesterol, HbA1c: glycated hemoglobin, DM: diabetes mellitus, HTN: hypertension

Variable	Double vessel PCI (n = 324)	Triple vessel PCI (n = 27)	Test statistic	p-value
Age, mean ± SD	61.23 ± 10.86	62.33 ± 9.09	t = 0.096	0.597
Age group, n (%)				
<45 years	25 (7.7)	1 (3.7)	Fisher’s exact test	0.936
45-54 years	61 (18.8)	4 (14.8)		
55-64 years	106 (32.7)	10 (37.0)		
65-74 years	101 (31.2)	10 (37.0)		
>75 years	31 (9.6)	2 (10.9)		
Gender, n (%)				
Male	264 (81.5)	23 (85.2)	Fisher’s exact test	0.632
Female	60 (18.5)	4 (14.8)		
BMI group, n (%)				
Normal weight	36 (11.1)	0 (0.0)	Fisher’s exact test	<0.001*
Overweight	74 (22.8)	0 (0.0)		
Obese	214 (66.0)	27 (100)		
Year of procedure, n (%)				
2019	47 (14.5)	6 (22.2)	Fisher’s exact test	0.758
2020	83 (25.6)	5 (18.5)		
2021	49 (15.1)	5 (18.5)		
2022	48 (14.8)	5 (18.5)		
2023	74 (22.8)	4 (14.8)		
2024	23 (7.1)	2 (7.4)		
Total cholesterol (mg/dL), median (IQR)	168.3 (125.1-216.8)	154.3 (135.8-186.1)	U = -1.019	0.308
Triglycerides (mg/dL), median (IQR)	174.55 (99.7-210.7)	163.2 (100.4-201.1)	U = -0.226	0.821
HDL-C (mg/dL), median (IQR)	43.77 (33.8-48.3)	40.0 (34.5-44.8)	U = -0.507	0.612
LDL-C (mg/dL), median (IQR)	120.7 (86.5-161.2)	92.5 (78.6-129.2)	U = -1.770	0.077
Diabetes mellitus, n (%)	188 (58.0)	17 (63.0)	χ² = 0.250	0.617
HbA1c (%), median (IQR)	6.80 (5.83-8.43)	7.46 (5.60-8.22)	U = -0.632	0.527
Prediabetes, n (%)				
Yes	76 (23.5)	6 (22.2)	χ² = 0.977	0.884
No	248 (76.5)	21 (77.8)		
Controlled DM, n (%)				
Yes	85 (26.2)	91 (25.9)	χ² = 0.008	0.648
No	239 (73.8)	260 (74.1)		
Uncontrolled DM, n (%)				
Yes	89 (27.5)	10 (37.0)	χ² = 1.127	0.288
No	235 (72.5)	17 (63.0)		
Hypertension, n (%)				
Yes	218 (67.3)	22 (81.5)	χ² = 2.323	0.127
No	106 (32.7)	5 (18.5)		

Patients aged ≥65 years were more likely to undergo multivessel PCI (41% vs 35%). In comparison, the proportion of younger patients (<45 years) was low in both groups, reflecting the low prevalence of extensive coronary involvement in younger ages. Gender distribution was similar across groups, with males representing the majority of cases in both single- and multivessel PCI. BMI demonstrated a marked association with disease extent; obese patients (BMI ≥30 kg/m²) accounted for 68.7% of the multivessel PCI group compared with 47.1% of the single-vessel group (p < 0.001). In contrast, normal-weight patients were less frequently affected by multivessel disease (Table 3).

**Table 3:** Baseline characteristics of patients undergoing single- versus multivessel PCI Statistical test used: chi-square test. * Significant p-value <0.05. BMI: body mass index, PCI: percutaneous coronary intervention

	Single vessel PCI (n = 978)	Multi vessel PCI (double + triple) (n = 351)	Test statistic	p-value
Age group, n (%)				
<45 years	110 (11.2)	26 (7.4)	χ² = 6.628	0.084
45-54 years	195 (19.9)	65 (18.5)		
55-64 years	331 (33.8)	116 (33)		
≥65 years	342 (35)	144 (41)		
Gender				
Female	177 (18.1)	64 (18.2)	χ² = 0.003	0.955
Male	801 (81.9)	287 (81.8)		
BMI group, n (%)				
Normal weight	175 (17.9)	36 (10.3)	χ² = 48.057	<0.001*
Overweight	342 (35)	74 (21.1)		
Obese	461 (47.1)	241 (68.7)		

In logistic regression analysis, advanced age and obesity remained significant factors associated with multivessel PCI after limited adjustment for multivessel coronary artery disease. Patients aged ≥65 years had nearly twice the odds of undergoing multivessel PCI compared with those <45 years (OR 1.90, 95% CI 1.17-3.09, p = 0.009), whereas the 45-54 and 55-64 age groups showed non-significant trends toward higher risk. Moreover, obese patients were more than two-and-a-half times as likely to have multivessel involvement as patients with normal weight (OR 2.62, 95% CI 1.77-3.88, p < 0.001). In contrast, overweight status was not associated with increased odds. Gender was not a significant predictor in the adjusted model (Table 4).

**Table 4:** Multivariable logistic regression analysis of factors associated with multivessel PCI (double + triple vessels) compared to single-vessel PCI * Significant p-value. Model adjusted only for age, sex, and BMI. Residual confounding from unmeasured clinical variables cannot be ruled out. OR:odds ratio, CI: confidence interval, BMI: body mass index, PCI: percutaneous coronary intervention

	Adjusted OR	95% CI	p-value
Age			
<45 years (reference)			
45-54 years	1.555	0.923-2.618	0.097
55-64 years	1.528	0.940-2.484	0.087
≥65 years	1.902	1.172-3.088	0.009*
Gender			
Female (reference)			
Male	1.158	0.831-1.612	0.386
BMI			
Normal (reference)			
Overweight	1.081	0.697-1.677	0.729
Obese	2.616	1.766-3.875	<0.001*

## Discussion

This retrospective study found that advanced age (≥65 years) and obesity (BMI 30-39.9 kg/m²) were significantly associated with angiographically confirmed multivessel coronary artery disease among patients undergoing PCI. These findings highlight the association between advancing age, obesity, and coronary atherosclerosis, which further underscores the need for elderly- and obese-focused comprehensive lifestyle changes, along with tailored medical management. In this study, only 27 patients underwent triple-vessel PCI, limiting the statistical power and precision of subgroup comparisons. Therefore, analyses comparing double- and triple-vessel PCI should be considered exploratory and interpreted cautiously.

In our study, patients with multivessel coronary artery disease undergoing PCI had been observed to have a higher mean age compared with single-vessel PCI. Similar findings have been reported by Batra et al. [10] in patients diagnosed with ST-elevation myocardial infarction and He et al. [12] in acute coronary syndrome patients. Moreover, the regression analysis revealed that patients aged ≥65 years were nearly twice as likely to have multivessel coronary artery disease compared with those aged <45 years. Previous studies showed that advancing age is considered a strong factor associated with atherosclerotic cardiovascular disease through the acceleration of coronary atherosclerosis via vascular mitochondrial dysfunction and inflammatory pathways [13,14], contributing to the progression from single-vessel to multivessel coronary artery disease. These findings highlight the importance of preventive measures for multivessel disease in elderly patients, along with medication and lifestyle changes.

Among patients aged <45 years, single-vessel coronary artery disease was more common than multivessel coronary artery disease. This is consistent with findings from Roth et al. [2] and Waziri et al. [15]. This is explained by studies examining clinical outcomes in patients undergoing PCI, which revealed that younger patients are characterized by better prognoses, shorter hospitalization durations, and lower mortality rates than their older counterparts [15,16]. These findings underscore the strong association between age and severity of coronary artery disease and further highlight the need for treatment strategies that are tailored to the patient’s age and risk profile.

Obesity is among the leading risk factors for the development and progression of atherosclerotic cardiovascular disease. Moreover, it is strongly linked to predictors of atherosclerotic cardiovascular disease, including type-2 diabetes mellitus and hypertension, as well as lipid profile abnormalities known as dyslipidemia [11]. Our study findings indicate that obese patients had approximately 2.6-fold higher odds of multivessel disease compared with their normal-weight counterparts, which is consistent with the current knowledge in that obesity is considered a risk factor for multiple cardiovascular diseases. However, contrasting findings have been reported by Ibrahim et al. [17], where obese patients were less likely to have multivessel disease in comparison to overweight and normal weight patients. It is worth noting that although obesity is a leading associated characteristic for coronary artery disease, it is associated with better clinical outcomes and reduced mortality rates after PCI, in what is known as the "obesity paradox" [18-20].

There were no significant differences among multivessel groups regarding lipid profile parameters; however, triple-vessel patients showed lower LDL-C levels. Contrasting findings have been reported by Tanaka et al. [21], where a non-significant increase in LDL-C levels was concordant with the number of vessel diseases. Diabetes mellitus increases the risk of developing multivessel coronary artery disease, along with less desirable outcomes following revascularization. Moreover, it is an independent risk factor for mortality in coronary artery disease, and it was found to be a stronger predictor than the extent of disease or the number of affected vessels [22]. Regarding the number of involved vessels, a study conducted by Arshad et al. [23] showed that the number of diabetic patients with double-vessel disease was slightly higher than triple-vessel diabetic patients, which is comparable to our findings. Moreover, uncontrolled diabetes is associated with increased risk of significant coronary atherosclerosis, leading to multivessel disease and high-risk coronary artery disease [24], which explains our findings of a slightly higher prevalence of uncontrolled diabetes in triple-vessel patients.

Strengths and limitations

This study is limited by its retrospective nature and dependence on existing clinical records, which may not capture other behavioral risk factors such as smoking, diet, or physical activity. Moreover, the findings may not be generalizable to other populations. Due to sparse counts in the triple-vessel category, vessel involvement was dichotomized to ensure model stability. Another major limitation of this study is the merging of BMI categories into broader groups for the regression analysis, which was done to avoid model overfitting. Because of the small number of triple-vessel PCI cases, subgroup analyses may have been underpowered and prone to unstable estimates. Therefore, comparisons between double- and triple-vessel PCI should be interpreted as exploratory. Additionally, patients with more extensive coronary artery disease may be more likely to undergo coronary artery bypass grafting rather than PCI, which may introduce potential treatment-selection bias in the study population. Although this approach improved statistical robustness, it may have reduced the ability to detect associations within individual BMI categories. Additionally, the exclusion of patients with incomplete procedural data may have reduced the statistical power and introduced selection bias if the excluded patients differed systematically from those included in the analysis. However, this study has several strengths, including a large sample size, which supports the reliability of the findings, and an extended study period, which minimizes bias.

Future directions

Future studies should implement prospective cohort designs and integrate lifestyle, medication, and genotype information to build a more comprehensive risk profile. This will lead to individualized treatment plans. Furthermore, innovations in PCI have expanded its role in the management of multivessel coronary artery disease, leading to improved patient outcomes and fewer complications. Implementation of procedural strategies such as radial artery access along with complete revascularization using PCI has several advantages, such as reduced bleeding and cardiovascular adverse effects, faster recovery times, shorter hospitalization duration, and better long-term survival rates [25]. Other novel approaches include the use of bioresorbable vascular scaffolds that dissolve completely after the treated vessel has healed [26], in addition to the implementation of intravascular imaging technologies, namely intravascular ultrasound and optical coherence tomography, for image guidance during PCI procedures, leading to improved decision-making, optimized PCI, and improved clinical outcomes [27]. Moreover, robot-assisted PCI has the potential to make coronary interventions more accessible to patients in remote and underprivileged areas [28].

## Conclusions

This retrospective study shows that obesity and advanced age were associated with multivessel coronary artery disease among patients undergoing PCI and that obese patients and patients aged ≥65 years were more likely to receive a multivessel intervention, which further supports the association of advanced age and obesity with cardiovascular conditions, such as coronary artery disease. Further research is needed to provide additional data from a Saudi cohort regarding the multivessel PCI group in obese and elderly patients.
